# People are essential to linking biodiversity data

**DOI:** 10.1093/database/baaa072

**Published:** 2020-11-27

**Authors:** Quentin Groom, Anton Güntsch, Pieter Huybrechts, Nicole Kearney, Siobhan Leachman, Nicky Nicolson, Roderic D M Page, David P Shorthouse, Anne E Thessen, Elspeth Haston

**Affiliations:** Biodiversity Informatics, Meise Botanic Garden, 1860, Meise, Belgium; Botanic Garden and Botanical Museum Berlin, Freie Universität Berlin, D-14195, Berlin, Germany; Biodiversity Informatics, Meise Botanic Garden, 1860, Meise, Belgium; Biodiversity Heritage Library Australia (Museums Victoria), VIC 3001, Melbourne, Australia; Independent Researcher, Wellington, New Zealand; Biodiversity Informatics, Royal Botanic Gardens, Kew, Richmond, TW9 3AE, UK; Institute of Biodiversity Animal Health & Comparative Medicine, University of Glasgow, Glasgow, G12 8QQ, UK; Biodiversity and Bioresources, Agriculture and Agri-Food Canada, Ottawa, ON, K1A 0C6, Canada; Oregon State University, Environmental & Molecular Toxicology, Corvallis, OR, 97331, USA; Herbarium Department, Royal Botanic Garden Edinburgh, Edinburgh, EH3 5LR, UK

## Abstract

People are one of the best known and most stable entities in the biodiversity knowledge graph. The wealth of public information associated with people and the ability to identify them uniquely open up the possibility to make more use of these data in biodiversity science. Person data are almost always associated with entities such as specimens, molecular sequences, taxonomic names, observations, images, traits and publications. For example, the digitization and the aggregation of specimen data from museums and herbaria allow us to view a scientist’s specimen collecting in conjunction with the whole corpus of their works. However, the metadata of these entities are also useful in validating data, integrating data across collections and institutional databases and can be the basis of future research into biodiversity and science. In addition, the ability to reliably credit collectors for their work has the potential to change the incentive structure to promote improved curation and maintenance of natural history collections.

## Introduction

Explicitly linking the entities of biodiversity research is a goal of 21st-century biodiversity informatics. These entities include taxa, taxonomic names, specimens, places, traits, molecular sequences and literature (1). Creating these links allow us to describe biodiversity, provide evidence for theories, support prediction, support reproducibility, give credit and ultimately underpin policy (2). Yet creating links confidently and ensuring that they remain stable and refer to the same thing remain a challenge. Some entities are more mutable than others; some are more ambiguous.

The cataloguing of specimens by taxon has always been problematic. Taxa are not stable indivisible entities; they are hypotheses of taxonomists who try to order the natural world and, to some extent, understand the process of evolution. For this reason, even an experienced taxonomist can find it hard to find a specimen in a collection, because different names and classifications are used. There are nevertheless powerful reasons for classifying collections by taxa, because taxonomy is one of the most pertinent use cases for physical access to specimens. Still, the use cases and options for digitized specimen data are much more varied (3).

People, unlike taxa, are unitary entities, and, while their names may change, the entities they refer to are finite and indivisible. The importance of people in many disciplines has long been recognized. For example, a book is a creative work. The key to finding, cataloguing and citing a book is the name of the author. Likewise, this is also true in the visual arts, dramatic arts and music. However, the people associated with the specimens have generally played a subsidiary role to that of the taxonomic identification, the geographical origin and even the date of collection.

We pay considerable attention to the data about people. We record significant dates in their lives, who they associated with, where they work, where they travel and many other facts of their life history. Scientists, who often are collectors and identifiers of specimens, also publish papers and books. These outputs are also well documented with dates, addresses and co-authors. Biographies are therefore well recorded and can be linked to the literature and specimens.

By making these links, biographies become a powerful tool for understanding the context of a specimen and cross-validating its data. For example, in a study of 20 million aggregated herbarium specimen records, 36% were evaluated to be in a duplication relationship (4). Most of these are duplicates in different herbaria and not linked to each other digitally. If these links were made, data from the collection event and the subsequent history of the specimen could be shared between collections, thereby reducing curational work and improving data quality. By comparing all of the metadata of people, collections, publications and specimens, we would have much greater confidence that the data are reliable. All of these connections help us understand the provenance of the specimen, what it has been used for and by whom, and this all helps us connect the underlying biological theory to the evidence.

## People in biological collection databases

It has been common practice for the names of people who have collected or identified specimens to appear as text on specimen labels. The database systems operated in natural history collections make it possible to store person names in a standardized and reusable manner. In some systems, the records for people include biographical data, such as date of birth, date of death and the taxonomic and geographic focus of the person. However, whilst this information has often been published in a wide range of digital and analogue resources, there has not been consistent use of identifiers. Names make poor identifiers because a person’s name can change, different people can have the same name, and names can be spelled in a variety of ways. A preferable solution would be to publish biographical data together so that local databases need only establish links to these jointly maintained resources that are designated by globally unique identifiers.

**Figure 1.**
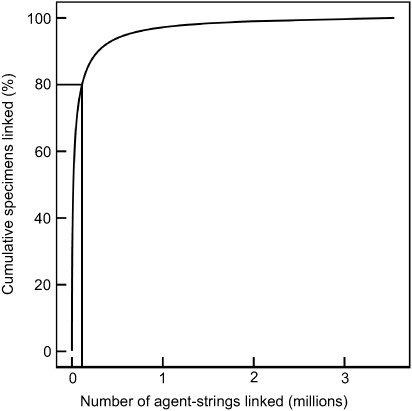
Cumulative percent of specimens associated with rank order of raw collector name strings. Collector name strings were parsed using the dwc_agent ruby gem, https://rubygems.org/gems/dwc_agent from 199M occurrence records downloaded from the Global Biodiversity Information Facility, https://doi.org/10.15468/dl.apmkx0 and are not reconciled or resolved to authoritative sources.

Figure 1 demonstrates that a large number of specimens can be linked rapidly to name strings. The distribution of specimens associated with people approximates to a power law (5). This means that if name strings for the 3% of most prolific people are connected to identifiers, then this will connect 80% of specimens. This also demonstrates that there is a long tail of less prolific people. These people are nonetheless important and will require specific attention over the long term.

## Current identifiers for biodiversity researchers

A number of person identifier systems exist, all with their own *raison-d’etre*, scope and properties. One of the first systems for biodiversity researchers was the Authors of Plant Names database published in 1992 by Brummitt and Powell (6). This assigned standardized abbreviations for all known authors of plant names. Similarly, Harvard University has an index of botanists (https://kiki.huh.harvard.edu/databases/botanist_index.html), including a persistent uniform resource locator for an individual botanist.

More widely applicable, the Virtual International Authority File (VIAF, http://viaf.org/) is an aggregator of authority files from major libraries. Its content is therefore mainly of authors of written works. However, there is overlap with scientific scholarly works. Each entry has a locally unique ID and a globally unique ‘Permalink’. A related resource is the International Standard Name Identifier (ISNI, http://www.isni.org/) that also incorporates VIAF.

The most recent identifier system is the ORCID ID (https://orcid.org/). ORCID is a not-for-profit organization set up to create unique identifiers for researchers. These identifiers are intended for living authors who are expected to curate their own entry.

Finally, Wikidata (https://www.wikidata.org) is becoming an increasingly important resource in the life sciences (7). While not specifically a person identifier system, Wikidata contains a wealth of information on people, both living and dead. Wikidata allows for the identification of people not under the scope of other person identifiers, such as those who are not published, but who are notable in other ways, such as being mentioned in the literature or collectors of specimens (8).

## The way forward

It is clear from the use of the existing identifier systems and the diversity of databases holding data about people that converging on a single identifier system is not possible. Therefore, a brokerage system is required to form an identifier network. ORCID is seen as an important part of the future identifier network, because it is used by the consent of the global scientific community and Wikidata can be used as the brokerage system to other identifiers.

Wikidata is importantly different from other identifier resources. It can be edited by anyone, which means that mistakes and omissions identified by a user can be immediately fixed by that user, thereby avoiding obstacles and delays caused by inflexible infrastructure. All data in Wikidata are published under a Creative Commons public domain dedication (CC0 https://creativecommons.org/publicdomain/zero/1.0/), meaning that there are no restrictions on what the data can be used for and how. Wikidata is also a multilingual database.

Wikidata has no authority of its own; it is a secondary source. It gains authority by being a broker of identifiers and by referencing authoritative data. Wikidata provides a wide variety of additional data and links, all of which can be used to validate, disambiguate and enrich data on people. For example, there are already large amounts of data on geographical units and organizations with which people can be effectively linked within the system. Furthermore, when individuals or projects contribute to Wikidata for their own benefit, their contribution also benefits the whole community.

In addition to the research benefits of being able to persistently and uniquely identify the people involved in the collection and curation of natural history specimens, there are several other benefits to science. For example, large-scale identification of people from collection metadata makes it tractable to assign professional credit to the collectors and curators, for work that is unlikely to be published directly. This is important because the current emphasis on publications and proposals for professional reward has the effect of deprioritizing the curation and maintenance of collections. Identifying authors of publications for the purposes of professional credit is common practice in science, whereas in collections management names and affiliations are frequently not recorded. Proper identifiers for people have the potential to change the sociology of science and research practices in the same way as the emphasis on publications has done for authors. Furthermore, by using common identification systems, we are creating a consistent information space that makes cross-collection inference possible for the first time (4).

People and scientific names are only some of the named entities on specimens. Geographic entities, such as political units and physical features of the landscape, are another example. There are explicit and implicit links to other specimens, literature, genetic sequence and species traits. However, the solidity and reliability of person data, if unique, persistent, resolvable identifiers for people were broadly implemented in natural history collections, make it a foundation upon which we can anchor the links of biodiversity research and build resources that help us access the wealth of biodiversity knowledge.

## To create the identifier network

- To gain credit for the whole corpus of their work, biodiversity scientists and collectors should register for an ORCID ID and provide at least one item of disambiguating biographical detail, such as an institutional affiliation. They should then associate their ORCID ID at every opportunity with their works, whether these are publications, observations, molecular sequences or specimens.
- In biodiversity collections, existing people identifiers should be included in collection management systems and attached to the specimen records. Institutions should contribute their published collector information to common biographical databases.
- Biodiversity infrastructures should support the use of person identifiers for living and dead people and standards development organizations should support person identifiers in their standards, including accommodating collecting teams and the order in which they are listed.
- Wikidata should be used as a brokerage system, enabling the linkage of identifiers from ORCID, VIAF, ISNI, HUH Index of Botanists, Authors of Plant Names and other commonly used identifier systems.
